# Do Family Obligations Contribute to Academic Values? The Mediating Role of Academic Efficacy

**DOI:** 10.3390/bs15091212

**Published:** 2025-09-05

**Authors:** Ciara S. Glover, Mayra Y. Bámaca, Kazumi Homma

**Affiliations:** 1 Department of Psychology, Georgia State University, Atlanta, GA 30302, USA; khomma@gsu.edu; 2 Psychological Sciences, University of California, Merced, CA 95343, USA; mbamaca@ucmerced.edu

**Keywords:** motivation, family obligations, family responsibilities, financial stressors, academic values, self-efficacy, young adult carer

## Abstract

Existing frameworks of task values have called for greater attention to contextual factors that inform decision-making. A critique of this research is a lack of attention to the cultural and situational milieu embedded in motivational theories. Investigating the development of academic values through obligations to the family and self-perceptions of academic ability adds to our understanding of the broader factors that drive student motivation in STEM. This paper explored the roles of family-related obligations associated with the motivational utility values of college STEM majors and the mediating role of academic efficacy. College students at two large ethnically diverse public research institutions shared their experiences in an initial survey as part of a larger longitudinal study on student adversity, motivation, and persistence in STEM (*N* = 1571, *Mage* = 20.41). The results revealed that academic efficacy weakens the roles of caregiving obligations on the perceived utility of their STEM major. The role of financial obligations to the family on students’ utility values operated indirectly through self-efficacy. Implications of these findings for future research are discussed.

## 1. Introduction

According to the Situated Expectancy Value Theory (SEVT), self-efficacy is a powerful predictor of academic goal setting, values, and persistence (Eccles & Wigfield, 2020; Wigfield & Eccles, 2023). The importance of academic self-efficacy continues throughout college (Chun et al., 2016). SEVT emphasizes the family’s role in how a child interprets and understands their abilities and performance (Eccles & Wigfield, 2020; Wigfield & Eccles, 2000). The situated framework highlights that these processes occur within specific contexts and situations. Scholars have called for increased attention to the contextual or “situated” aspects of academic motivational processes (Matthews & Wigfield, 2024). Obligations to the family are a context that may influence how students perceive their academic abilities and may ultimately affect their academic attitudes.

Our research questions are guided by the Situated Expectancy-Values Theory (SEVT, Eccles & Wigfield, 2020). We focus on self-perceptions as crucial factors in developing academic values. Family responsibilities are framed within the child’s interpretation and perceived control over those experiences. How these obligations are appraised influences perceived academic goals and self-schemas, which ultimately shape the student’s academic values (Eccles & Wigfield, 2020). In an investigation across 59 countries, family caregiving expectations predicted higher achievement among those with an interdependence orientation to motivation, compared to those in individualistic societies (Li et al., 2021). It is plausible that societal and cultural contexts inform student appraisal of family responsibilities.

### 1.1. Family Obligations

Student caregivers are a growing but largely underrecognized population by educational institutions (Armstrong-Carter et al., 2022; Chevrier et al., 2022). For these students, family responsibilities may indirectly contribute to the perceived usefulness of their college STEM major and subsequent degree attainment through changes in perceptions of their academic abilities. Therefore, it is important to go beyond the role of academic settings in examining the academic success of college-attending young adults. Bronfenbrenner’s Bioecological Model (Bronfenbrenner & Morris, 2006) underscores the role that family contextual realities have on individuals’ development and adaptation. Amongst young adults, family background characteristics and parenting (Weiser & Riggio, 2010), emotional, financial, and instrumental support (Osuji et al., in press; Wiggins, 2025), family adversity (e.g., health problems; Charles et al., 2004), and family obligations (Fuligni & Witkow, 2004) have been associated with academic-related outcomes. Synonymous with *family responsibilities* in the current paper, *family obligations* can be conceptualized as contextual demands that involve continued physical and/or mental effort (Ten Brummelhuis & Bakker, 2012). For college students, combined school and family responsibilities (e.g., caregiving and financial support to the family) can contribute to stress and adversely impact school performance (Olson, 2014).

The focus on family responsibilities’ role in college outcomes is necessary, as today’s U.S. college students represent a more diverse student population that navigates multifaceted tasks, including family responsibilities (Armstrong-Carter et al., 2022; Olson, 2014). About 50% of domestic college students are non-White (Kim et al., 2024), slightly more than 50% have independent status (e.g., age 24 or older, having dependents of their own; Reichlin et al., 2018), and about 1/3 are the first generation in their family to ever attend college (vis-à-vis continuing-generation college student). These factors have been associated with financial vulnerability (Johnson, 2019) and fewer family resources (Armstrong-Carter et al., 2022; LeBouef & Dworkin, 2021). Moreover, during the COVID-19 pandemic, financial difficulties for students and their families were widespread (Ramirez et al., 2023), which could have increased caregiving obligations for students. Ramirez et al. (2023) found that during the pandemic, college students of diverse backgrounds reported a high prevalence of family job loss or reduction in work hours: 87% of Latiné, 75% of other underrepresented, 57% of White, and 54% of Asian American. Moreover, Marshall and Naumann (2024) reported that 45.5% of college participants in their study reported caregiving for someone. These figures suggest that family-life events may lead to college students’ increased family obligations (e.g., caregiving and financial) and spill over to their academic experience.

Family obligations have been conceptualized as values and as responsibilities to help; these two differentially contribute to young people’s outcomes. For example, Fuligni et al. (1999) define family obligations as the degree to which family members believe it is important to help the family and consider the family’s needs when making decisions (Fuligni et al., 1999). Alternatively, Sy and Romero (2008) describe family obligations as helping behaviors that young people engage in to assist the family, including caregiving for siblings, providing financial support, and acting as cultural brokers (e.g., translating for parent–child conferences or doctor’s appointments). Synonymous with the current paper, the latter conceptualization taps into the degree to which young people experience demands to help the family in areas such as caregiving. We refer to this as *family responsibilities.* Much past research suggests that engaging in family responsibilities can be a stressful experience, impacting adaptation; however, the extent to which these behaviors are viewed as burdensome remains unclear. The current study examines perceptions of caregiving and financial obligations as detracting from academic coursework.

Evidence shows that within studies on family obligations, values and helping behaviors are independently associated with academic outcomes. Valuing family obligations is generally associated with positive outcomes (cf., Toyokawa & Toyokawa, 2019). For example, Fuligni (2001) found that adolescents who reported more family obligation values were also more likely to report a higher value for academic success and perceived greater utility for education, math, and English than those with low levels of family obligations. Similar work has found that for students with low and high school GPAs, family obligation values were associated with greater educational persistence (i.e., being enrolled in college) (Fuligni & Pedersen, 2002). Overall, these findings suggest that values that emphasize the importance of helping the family may function as motivators for students’ persistence in college as well as their perceptions that having more education is useful (i.e., academic utility value).

On the other hand, responsibilities to help the family, in expected and unexpected ways, can be a significant hurdle for students’ academic success (Arana et al., 2011). Two family responsibilities that have received attention in the literature are caring for a family member (caregiving responsibilities) and contributing financially to parents (financial responsibilities). There is limited work on students’ perceptions of family responsibilities as barriers to higher education but a growing body of research on adolescent caregivers reveals interruptions to their learning and academic performance (Siskowski, 2006). In general, existing studies with young adults assess the presence or frequency of caregiving and its effect on young adults’ mental health and college experiences; study findings support the premise that helping the family may be perceived as burdensome, as it has been linked to poor outcomes. For example, caring for elders or ill family members was associated with depressive symptomatology, anxiety, and lower academic performance (Armstrong-Carter et al., 2022). Providing financial assistance to the family may be fulfilling for individuals who are from cultural backgrounds that place great emphasis on family obligations and assistance (Telzer et al., 2010). However, this responsibility can also create undue stress and affect students’ engagement in college. For example, in a sample of young adults from East Asian, Filipino, Latin American, and European backgrounds, students who provided financial support to their families were less likely to enroll in any degree or persist in college (i.e., not currently attending a 2- or 4-year degree) (Fuligni & Witkow, 2004).

Overall, this research suggests that contributing to family responsibilities (i.e., caregiving and financial support) that are perceived as stressful may be linked to less optimal academic outcomes. Past studies have focused on frequency of family responsibilities. Frequency metrics do not account for differences in how people feel about these obligations. frequency of the responsibilities. Young adults’ *evaluation* of their responsibilities to their family and its potential impact on their academic values has received little attention. In addition, less is known about the mechanisms by which this association takes place.

### 1.2. Academic Efficacy as a Mediator

Drawing from general self-efficacy, academic efficacy refers to belief in one’s perceived abilities to accomplish school tasks (Bandura, 1997; Pajares, 1996). We propose that in college, students may perceive family responsibilities as burdensome, and this can function as a contextual backdrop that may lead to a (re)formulation of self as academically inefficient, which, in turn, can influence students’ views on the utility of education. That is, students’ perceptions of family obligations as challenging or burdensome may affect students’ perceptions of self (i.e., low academic self-efficacy), which can predict low perceptions of education utility value. Although not specific to family obligations, taxing experiences such as financial stress were linked to lower academic self-efficacy among college students (White & Perrone-McGovern, 2017). Moreover, other stressors have been directly and indirectly associated with academic outcomes via academic self-efficacy. Specifically, Chun et al. (2016) found that lower levels of acculturative stress predicted more academic self-efficacy, which predicted students’ GPA among Latiné college students. In another study with adolescents in China, self-efficacy partially mediated the association between school stress and school burnout. School stress was associated with lower levels of self-efficacy and higher levels of academic burnout. In turn, self-efficacy was related to lower levels of academic burnout (Jung et al., 2015).

The relationship between parents and children significantly influences their beliefs about their abilities. These beliefs can impact both immediate outcomes, such as motivation, and long-term outcomes, like degree attainment and overall performance. Research has shown that academic self-efficacy serves as a mediator between the quality of parent–child relationships and academic performance during college (Llorca et al., 2017). Although studies have explored how family relational quality affects college students’ academic success, there is limited research on how young adults’ obligations to their families relate to their self-efficacy, which in turn may shape their academic values.

### 1.3. Study Rationale

Familial experiences, even during the college years, can inform self-beliefs about academic abilities. Past research notes that these academic self-perceptions can inform motivational values; of interest in the current study are students’ attitudes about how useful their STEM degree will be. Previous research suggests that family responsibilities are stressful to young adult development when they occur frequently. However, frequency metrics do not consider variations in cultural perspectives. There is limited understanding of how caregiving is perceived as a burden, especially among college students from diverse backgrounds who manage multiple demands in their lives. The ways in which family obligations may detract from coursework are explored in the current study.

Considering the past research that has linked family responsibilities to poorer academic experiences of college students, it is expected that similar associations would emerge; caregiving obligations and financial responsibilities would be associated with less endorsement of the utility of the STEM degree. Our second hypothesis was that family obligations would be related to lower academic efficacy. We further expected that efficacy would be associated with higher utility values. Finally, we hypothesized that students’ academic self-efficacy would attenuate the link between obligations to family and perceptions of the usefulness of their degree (utility values).

Some work has shown that caregiving is higher among female and Latiné college students (Ramirez et al., 2023; Siskowski, 2006). Research further reveals that college degree attainment odds were lower among young adult carers in the UK and Germany, as were the odds of starting employment; these associations were more pronounced for female young adult carers (King et al., 2023). SEVT was developed, in part, to explain the mechanisms, like socialization, that inform gender differences in motivation and choice of STEM activities (Bembenutty, 2008; Wigfield & Eccles, 2000); there is also growing attention to the cultural and racial contexts that these choices are situated in (Lawson & Fong, 2024; Matthews & Wigfield, 2024; Robinson & Shankar, 2025). Given the existing research that links demographic characteristics (e.g., gender, race) to caregiving obligations and financial contributions to the family, we account for these variables in the model.

## 2. Materials and Methods

### 2.1. Materials

Original survey data came from Time 1 of a three-year mixed-methods study on the impact of various adversities on persistence in college-level STEM programs at two large public research universities in the southeast of the US that each enrolled over 50,000 students. Universities were Minority Serving Institutions. Eligibility was open to those enrolled in a STEM or pre-STEM major who were Freshmen (45.77%), Sophomores (30.78%), or Juniors (23.4%). Human subjects approval was obtained for the study and participant consent was collected. The larger multisite, mixed-method, longitudinal study examined the association between student adversity, motivation, and persistence in their STEM major. A total of 1760 undergraduate students enrolled in the larger study. The current study consisted of *N* = 1571 (male = 28.8%, female = 68.4%, non-binary = 2.8%), ages 20–36 (*M* = 26). The sample was ethnically and racially diverse (African American/Black students = 29.0%; White students = 9.2%; Asian students = 23.2%; Latinx students = 34.5%; Native American/Hawaiian/Alaskan Native students = 0.3%; biracial/multi-racial students = 3.5%; other students = 0.3%). For the current study, we used data from Time 1, collected from 2023 to 2024 via online surveys. We cleaned the data in SPSS (version 29.0) and read and analyzed it in R Studio (version 2023.12.0+369), using the lavaan, semPlot, and the sjPlot packages.

### 2.2. Methods

We tested our hypothesized theoretical model using structural equation modeling (SEM). While there are various ways to examine model fit, studies on SEM recommend looking at three different types of model fit indices, especially CFI (an incremental index), RMSEA (a parsimonious index), and SRMR (an absolute index). The literature discusses that critical values for these indices are CFI = 0.95, RMSEA = 0.06, and SRMR = 0.08, respectively (Hu & Bentler, 1999). To test the statistical significance of path coefficients—direct effects, indirect effects, total indirect effect, and total effect—the current best practice is to conduct inferential tests of these effects using bootstrapped 95% confidence intervals and Maximum Likelihood (ML) as an estimation method. Therefore, we applied this standardized procedure of SEM to our data analysis.

#### 2.2.1. Model

**Figure 1:**
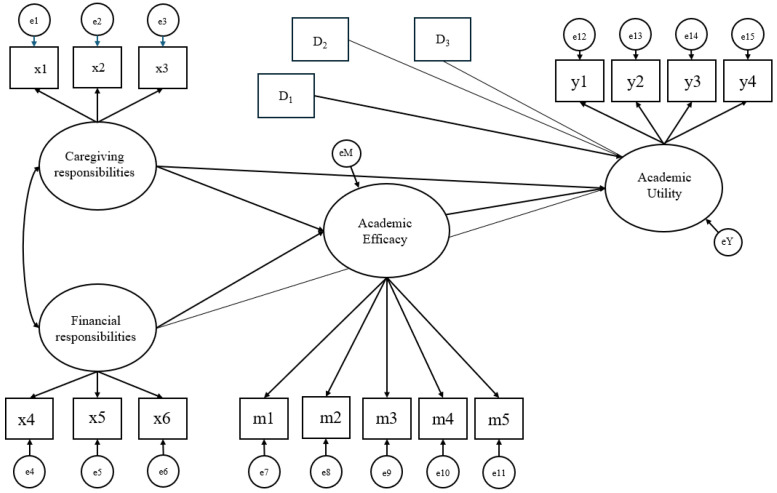
Measurement Model of Caregiving Responsibilities, Financial Responsibilities, Academic Efficacy, and Academic Utility.

We translated our hypothesized theoretical model into a one-mediator model with two predictors (Hayes, 2022), where the dependent variable is academic utility, the first predictor is caregiving responsibilities, the second predictor is financial responsibilities, and the mediator is academic efficacy. In this model, the effects of two predictors on the dependent variable occur through one mediator (Figure 1). Family responsibilities were used interchangeably with family obligations throughout the paper.

#### 2.2.2. Variable List

The respective variables are explained below:

1. Dependent Variable

First, the dependent variable was Academic Utility (Y), a latent variable informed by Conley (2012) and Eccles and Wigfield (1995). This variable was an ordinal variable comprised of four 5-point Likert scale items (Cronbach’s α = 0.883; CFI = 0.986; RMSEA = 0.125, 90% CI = 0.098, 0.154; SRMR = 0.018).

2. Predictors

Second, we included two predictors in our hypothesized model: Caregiving Responsibilities (X1) and Financial Responsibilities (X2). These predictors were original variables grounded in our observations. For instance, caregiving responsibilities facing emerging adults are multi-dimensional; they consist of various hardships associated with parents, partners, and children/siblings. In this study, these predictors were treated as latent variables. Caregiving Responsibilities (X1), non-financial, was associated with three observed indicators (Cronbach’s α = 0.875; CFI = 1.000; RMSEA = 0.000, 90% CI = 0.000, 0.000; SRMR = 0.000), while Financial Responsibilities (X2) was associated with three observed indicators (Cronbach’s α = 0.939; CFI = 1.000; RMSEA = 0.000, 90% CI = 0.000, 0.000; SRMR = 0.000). These indicators consisted of 5-point Likert scale items (1 = *Strongly Disagree*, 5 = *Strongly Agree*). A sample item for Caregiving Responsibilities (X1) is “Caregiving for a parent makes it difficult to focus on school (e.g., attend class, study, etc.).” Additionally, a sample item for Financial Responsibilities (X2) is “I feel stressed about not being able to save money because my family members ask me to help them meet their basic needs unexpectedly.” Higher scores reflected greater responsibilities.

3. Mediator

Third, Academic Efficacy (M) was assessed with 5-items from the Motivated Strategies for Learning Questionnaire (Pintrich et al., 1991; Midgley et al., 2000). This construct was treated as a latent variable comprised of 5 observed indicators (Cronbach’s = 0.857, CFI = 0.956; RMSEA = 0.136, 90% CI = 0.119, 0.154; SRMR = 0.003).

4. Covariates

Finally, we added three binary covariates (D1, D2, and D3) to this model. First, we added GENDER (D1) as a binary covariate (*Non-Female* = 0, *Female* = 1). The Non-Female category consists of male students and non-binary students. Second, we added a student’s racial-ethnic background as D2 (*Non-African American* = 0, *African American* = 1) and D3 (*Non*-Latiné = 0, Latiné = 1). In this study, we called these covariates Gender, African American/Black, and Latiné, respectively.

#### 2.2.3. Equations

1. Measurement Model

Figure 1 visually explains the measurement model. We treated all variables in the model as latent variables, except for covariates. To identify this model, the means and variances of these latent variables were fixed to 0 and 1, respectively.

2. Structural Equation Model

The structural equation model consists of the following equations:

M = a_2_·X1 + b_2_·X2                 

Y = a_1_·X1 + b_1_·X2 + c_1_·M + d_1_·D1 + d_2_·D2 + d_3_·D3

where M is the mediator, Y is the dependent variable, X1 is the first predictor, X2 is the second predictor, D1, D2, and D3 are covariates, a_1_ is the direct effect of X1 on Y, a_2_ is the effect of X1 on M, b_1_ is the direct effect of X2 on Y, and b_2_ is the effect of X2 on M. In addition, we calculated direct effects, specific indirect effects, and total effects to answer the respective research questions.

## 3. Results

### 3.1. Descriptive Statistics and Correlation Analysis

#### 3.1.1. Descriptive Statistics

We ran an analysis of variance (ANOVA) to see if there were mean differences in students’ (a) caregiving and financial responsibilities and (b) academic efficacy across groups (i.e., African American/Black students and Non-African American/Black students, Latiné students and non-Latiné students, and female students and non-female students (i.e., male students and non-binary students), respectively. The reference categories were Non-African American, non-Latiné, and non-female, respectively. The results show that African American/Black students may have had more family obligations than Non-African American/Black students (African American/Black: *M* = 5.83; Non-African American/Black: *M* = 4.64; F = 49.68, *df* = 1, *p* < 0.00). Non-female students (Female: *M* = 6.58; Non-Female: *M* = 7.38; F = 19.37, *df* = 1, *p* < 0.00) may have had more family obligations than female students, respectively.

#### 3.1.2. Correlation Analysis

We examined correlations among all variables included in the model (*N* = 1571). Results indicated significant associations among key variables in our SEM model (see Table 1).

### 3.2. Model Fit and Parameter Estimates

**Figure 2:**
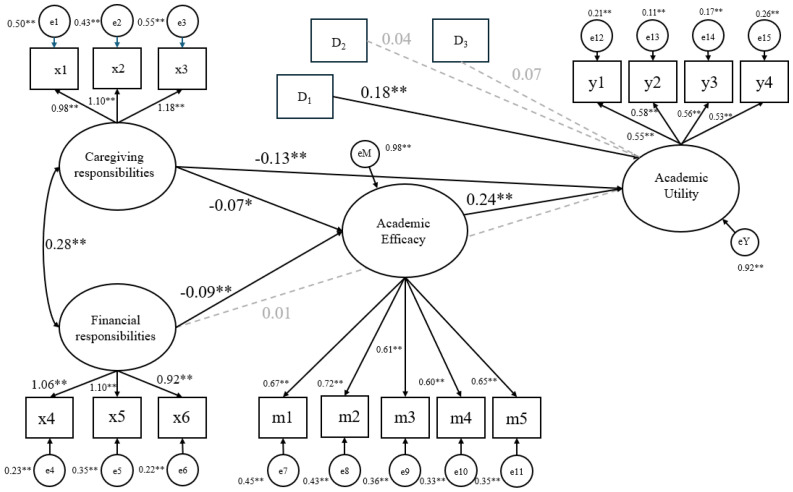
Mediation Model of Academic Efficacy in the Relationship Between Caregiving and Financial Responsibilities and Academic Utility. Note. ** *p* < 0.001. * *p* < 0.05. Dotted lines are not statistically significant.

We examined model fit indices and parameter estimates using the lavaan output. Given that our model had three covariates that potentially influence model fit, we ran and examined model fit indices without these covariates. The results indicated that our hypothesized theoretical model fitted the data pattern reasonably well (CFI = 0.97; RMSEA = 0.04, 90% CI = 0.4, 0.05; SRMR = 0.04). We then examined parameter estimates. For bootstrapping, we applied 10,000 iterations. The path diagram with standardized estimates overlaid is shown in Figure 2. The results are also summarized in Table 2.

### 3.3. Summary

With these results, our answers to respective research questions are summarized below:

**RQ1:**  *Are family responsibilities (i.e., caregiving and financial) associated with lower academic efficacy and utility values?*

Model results provided partial support for RQ1. Specifically, caregiving responsibilities were negatively associated with students’ academic utility (γ = −0.130; *p* < 0.000). However, financial responsibilities were not directly associated with academic utility (γ = 0.007; *p* = 0.820).

**RQ2:**  *Are family responsibilities associated with lower academic efficacy?*

Results indicated that reporting family caregiving and financial responsibilities were negatively associated with students’ academic efficacy (γ = −0.073, *p* = 0.018; γ = −0.094, *p* = 0.005, respectively).

**RQ3:**  *Is academic efficacy associated with academic utility values?*

Findings indicated that students’ academic efficacy had the greatest positive contribution on students’ perceptions of academic utility (γ = 0.238; *p* = 0.000). Its direct effect was much greater than the negative effect of caregiving responsibilities on a student’s academic utility (γ = −0.130; *p* = 0.000). Moreover, as stated above, financial responsibilities were not significantly associated with students’ academic utility.

**RQ4:**  *Does academic efficacy mediate the association between family responsibilities and academic utility values?*

Tests of mediation revealed that academic efficacy mediated the negative effect of family responsibilities, particularly of caregiving, on students’ academic utility. While caregiving responsibilities directly and negatively affected students’ utility value (γ = −0.130; *p* = 0.000), this negative effect diminished when mediated by students’ academic efficacy (X1 × M = −0.017, *p* = 0.028; X2 × M = −0.022, *p* = 0.010).

In summary, results partially supported our hypotheses such that perceived academic ability (i.e., academic efficacy) significantly weakened the negative effects of family responsibilities, particularly of caregiving responsibilities, on academic utility.

## 4. Discussion

The aim of this study was to explore how family obligations impact the formation of academic values among STEM majors. We anticipated that young adults who care for loved ones or contribute financially to their families might find it more challenging to focus on their coursework, leading them to value their STEM degree less. Our findings partially supported this expectation: young adults who felt distracted by caregiving responsibilities reported a lower valuation of their STEM major. This relationship underscores the importance of higher education programs that strengthen academic efficacy for students with significant family obligations.

Further, the findings reveal that when students financially support their families in stressful ways, it is associated with a lower perceived ability to engage in academic work. Moreover, caregiving that detracted from coursework was associated with lower academic efficacy. The results support existing literature (Jung et al., 2015; King et al., 2023; Xue et al., 2023). The current findings add to our understanding of how responsibilities that detract from coursework inform self-perceptions of one’s academic abilities, which in turn, inform values about the usefulness of one’s STEM degree.

Preliminary analyses revealed that caregiver obligations and efficacy differed only by gender (lower for females). Financial responsibilities were higher for African American students and non-Latiné students. The latter may reflect the responsibilities endorsed by African American students. We recognize that these associations differ from the existing literature, where females and Latiné students report more family obligations (King et al., 2023; Ramirez et al., 2023). It is plausible that differences in our study reflect the broader structural barriers experienced by families independent of demographic characteristics. The larger context that shaped responses may also include inflation and related pandemic barriers (Ramirez et al., 2023) which possibly changed the relational norms reported in prior literature.

### 4.1. Developmental Context

The study also makes important contributions within the educational literature by taking a person-context approach to better understand the factors implicated in individuals’ expectancy values. We add to the small body of research that addresses how situated factors inform academic values in the young adult years. Further, our work enhances an understanding of family obligations as demands that appear to interfere with academic domains during the college years.

Our findings build on studies that show that responsibilities to one’s family that co-exist with family reciprocity, autonomy support, or interdependency beliefs, promote resilience among minoritized college students (Chen & Wong, 2014; Jimenez et al., 2022; Wiggins, 2025). We developed a measure of financial obligation responsibilities to the family for the project, as existing measures did not adequately capture the perceived autonomy and actual contributions of financial support made by young adults. Overall, the contribution of the family responsibility measures in this study is that these align with the developmental context of young adulthood.

Future research on the role of family obligations during young adulthood should consider the degree to which these obligations may interfere with important developmental tasks in young adulthood. In contrast to the family obligations of younger children, young adults’ family obligations may reflect a level commensurate with their independence and ability to help. At the same time, these obligations can inform their developmental needs for autonomy and perceived competence during the young adult stage. Our findings suggest that during young adulthood, the responsibilities associated with family obligations diminish students’ perceptions of academic ability, which in turn informs their beliefs about the utility of their STEM degrees.

### 4.2. Limitations and Future Studies

We used cross-sectional data from the initial time point of a longitudinal study. Mediation can be tested with cross-sectional data provided it is grounded in theory, as was the case in our study. Additionally, we treated gender and race as binary variables due to modeling limitations, whereas the original variables were categorical. Future research may replicate these findings across time to examine how efficacy is shaped by family obligations throughout college, and in turn, contribute to the development of utility values. Future studies may further build on the foundation of our findings by investigating how the development of utility values is associated with other motivational values and academic decisions (e.g., degree attainment). Next steps for the larger project from which these data derive can include the use of student interviews and alternative models that consider demographic and behavioral moderators of family obligations and/or financial responsibilities. Lastly, in addition to considering the family-school interface, future research on young adults enrolled in college can examine other spheres such as the work–school interface, given that during this period of development, many students are navigating family, school, work, and social responsibilities, which may all play a role in college completion.

Although our focus on two universities may be considered a limitation of the study, we believe it is a strength that the data encompassed student bodies at two large public research institutions that were also Minority Serving Institutions.

## Figures and Tables

**Table 1:** Descriptive Statistics and Correlation Analysis.

	1	2	3	4	5	6	7
**1**	--	0.25 **	−0.10 **	−0.14 **	−0.08 **	−0.03	0.04
**2**	--	--	−0.10 **	−0.06 *	−0.05	0.17 *	−0.07 **
**3**	--	--	--	0.20 **	−0.07 *	−0.04	0.04
**4**	--	--	--	--	0.07 **	0.00	0.03
**5**	--	--	--	--	--	0.06 *	0.15 **
**6**	--	--	--	--	--	--	−0.46 **
**Mean**	6.80	5.01	21.10	17.66		--	--
***SD***	3.48	3.18	3.57	2.48			
***Range***	3–15	3–15	5–25	4–20	--	--	--

Note: Listwise deletion was applied to calculate the sample size. Full-information maximum likelihood (FIML) was unavailable due to modeling limitations in R; FIML is not compatible with mediation models with boostrapping. Given the conceptual nature of the race variable, we considered research ethics and did not apply multiple imputation to respect respondents’ self-reported racial identity. ** *p* ≤ 0.01 level (2-tailed). * *p* ≤ 0.05 level (2-tailed). 1 = Caregiving Responsibilities (X1); 2 = Financial Responsibilities, (X2); 3 = Academic Efficacy (M); 4 = Academic Utility (Y); 5 = Gender (D1); 6 = African American/Black (D2); 7 = Latiné (D3).

**Table 2:** Academic Efficacy as a Mediator.

Effect	Estimate	*SE*	95% CI		*p*
			LL	UL	
Direct effects on Academic Utility (Y)					
Caregiving Responsibilities	−0.130	0.032	−0.195	0.071	0.00
Financial Responsibilities	0.007	0.031	−0.054	0.067	0.82
Academic Efficacy (M)	0.238	0.037	0.167	0.314	0.000
Gender (D_1_) ^1^	0.176	0.063	0.054	0.299	0.005
African American/Black (D_2_) ^2^	0.039	0.075	−0.108	0.188	0.598
Latinx (D_3_) ^3^	0.071	0.063	−0.052	0.196	0.260
First paths of mediation					
Effect of X_1_ on M (_a2_)	−0.073	0.031	−0.135	−0.013	0.018
Effects of X_2_ on M (_b2_)	−0.094	0.034	−0.161	−0.030	0.005
Indirect effects					
X_1_ × M ^4^	−0.017	0.008	−0.034	−0.003	0.028
X_2_ × M ^5^	−0.022	0.009	−0.041	−0.007	0.010
Total indirect effects	−0.040	0.011	−0.063	−0.021	0.000
Total effect	0.361	0.134	0.102	0.626	0.007

Note: ^1^ Non-Female versus Female (reference category = Non-Female). ^2^ Non-African American versus African American (reference category: Non-African American). ^3^ Non-Latiné versus Latinx (reference category: Non-Latiné). ^4^ The indirect effect of caregiving responsibilities and academic efficacy on academic utility. See Figure 1 and Figure 2. ^5^ The indirect effect of financial responsibilities and academic efficacy on academic utility 1).

## Data Availability

The raw data and code are not openly available but may be available upon request from the corresponding author.
